# Supplementary material for: Re-Emergence of Salmonellosis in Hog Farms: Outbreak and Bacteriological Characterization
Source: Microorganisms. 2021 Apr 27;9(5):947. doi: 10.3390/microorganisms9050947 (PMC8146755; doi:10.3390/microorganisms9050947)
Supplement: Supplementary file 1 [file microorganisms-09-00947-s001.zip › Figure S2.pdf]

Dendrogram of monophasic variant of *S.* Typhimurium isolates according to pulsotype, outbreak location, and antimicrobial resistance profile

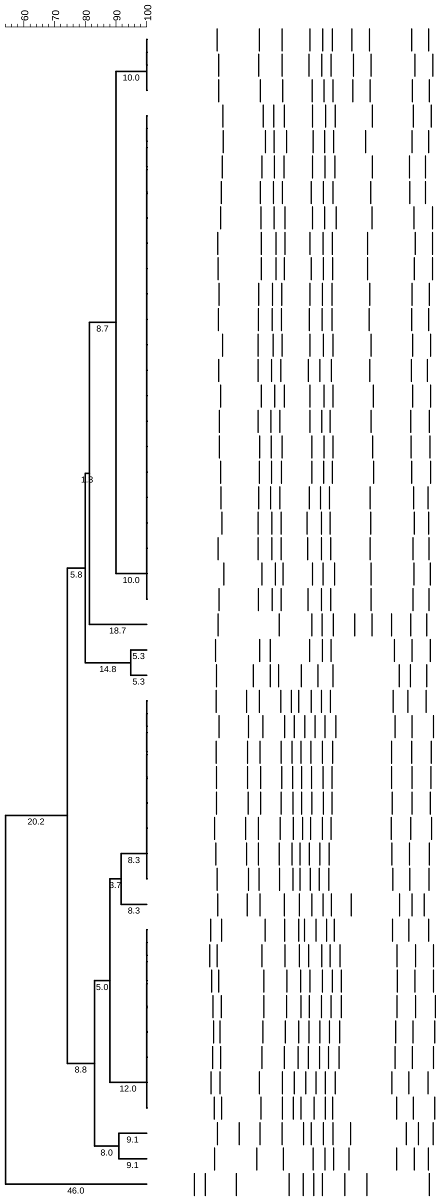

State	Number of Isolates	Antimicrobial Resistance
SC	1	COL, DOX, ENR, FFC, GEN, MAR, STR, TET
MG	1	CIP, COL, DOX, ENR, FFC, GEN, LSC, MAR, NOR, STR, SXT, TET
SC	1	CIP, COL, DOX, ENR, FFC, GEN, MAR, NOR, TET
MG, MT	2	CIP, COL, DOX, ENR, FFC, GEN, LSC, MAR, NOR, STR, SXT, TET
MG	1	CIP, DOX, ENR, FFC, GEN, MAR, NEO, NOR, STR, SXT, TET
MG	1	COL, DOX, ENR, FFC, GEN, MAR, NEO
MG	1	CIP, COL, DOX, ENR, FFC, GEN, MAR, NOR, STR, SXT, TET
SC	1	CIP, COL, DOX, ENR, FFC, GEN, LSC, MAR, NEO, NOR, STR, SXT, TET
MT	1	CEF, CIP, COL, DOX, ENR, FFC, FOS, GEN, MAR, NOR, STR, SXT, TET
MG	2	COL, DOX, ENR, FFC, GEN, LSC, MAR, STR, SXT, TET
SC	1	COL, DOX, ENR, FFC, GEN, MAR, TET
SC	1	CIP, COL, ENR, FFC, GEN, MAR, STR, TET
MG	1	CIP, COL, DOX, ENR, FFC, GEN, MAR, NOR, STR, SXT, TET
RS	1	ENR, FFC, GEN, MAR, STR, SXT
PR	1	CIP, DOX, ENR, FFC, GEN, MAR, NOR, STR, SXT, TET
MG	1	CIP, DOX, ENR, GEN, MAR, NOR, STR, SXT, TET
MG	1	CIP, COL, DOX, ENR, FFC, GEN, LSC, MAR, STR, SXT, TET
MG, SP	4	CIP, COL, DOX, ENR, FFC, GEN, MAR, NOR, STR, SXT, TET
MG	1	CIP, DOX, ENR, FFC, GEN, LSC, MAR, STR, SXT, TET
MG	2	CIP, COL, DOX, ENR, FFC, GEN, MAR, NOR, STR, SXT, TET
SC	1	CEF, CIP, COL, DOX, ENR, FFC, GEN, MAR, TET
MG	1	CEF, CIP, DOX, ENR, FFC, GEN, MAR, NOR, STR, SXT, TET
SC	1	CIP, COL, ENR, FFC, GEN, MAR, NOR, TET
PR	1	CEF, CIP, COL, DOX, ENR, FFC, GEN, LSC, MAR, STR, SXT, TET
RS	1	CEF, COL, SXT, TET
SC	1	CIP, COL, DOX, ENR, FFC, GEN, MAR, TET
DF	1	COL, DOX, ENR, FFC, GEN, NEO, STR, TET
SC	1	COL, DOX, GEN, TET
SC	1	CEF, COL, DOX, FFC, GEN, NEO, STR, TET
SC	1	COL, DOX, FFC, GEN, NEO, TET
SC	1	DOX, GEN, TET
MG	1	COL, DOX, TET
RS	1	DOX, ENR, GEN, LSC, TET
SC	1	COL, FFC, GEN, NEO, TET
SC	1	COL, DOX, FFC, GEN, LSC, SXT, TET
SP	1	COL, DOX, ENR, TET
MT, SP	2	COL, DOX, FFC, GEN, STR, TET
SC	1	COL, DOX, FFC, GEN, STR, TET
SC	2	COL, FFC, GEN, NEO, STR, TET
SC	1	COL, DOX, FFC, GEN, NEO, STR, TET
SC	1	COL, DOX, GEN, TET
MG, SC	2	COL, DOX, FFC, GEN, NEO, STR, TET
MG	1	DOX, ENR, FFC, GEN, MAR, SXT, TET
SC	1	CIP, COL, ENR, FFC, GEN, MAR, SXT, TET
SP	1	CIP, COL, DOX, ENR, FFC, GEN, MAR, SXT, TET
MG	1	COL, DOX, FFC, SXT, TET
