# Supplementary material for: Re-Emergence of Salmonellosis in Hog Farms: Outbreak and Bacteriological Characterization
Source: Microorganisms. 2021 Apr 27;9(5):947. doi: 10.3390/microorganisms9050947 (PMC8146755; doi:10.3390/microorganisms9050947)
Supplement: Supplementary file 1 [file microorganisms-09-00947-s001.zip › Figure S1.pdf]

Dendrogram of 130 *Salmonella* isolates according to pulsotype, outbreak location, serovar antimicrobial resistance profile

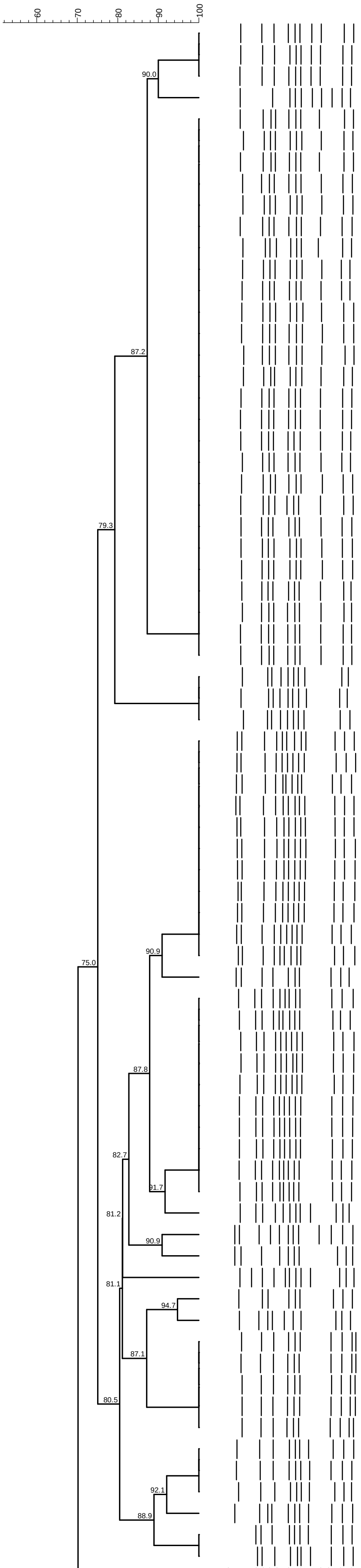

Key	State	Serovar	Antimicrobial Resistance	Sequenced
1585	MG	Monophasic	CIP, COL, DOX, ENR, FFC, GEN, LSC, MAR, NOR, STR, SXT, TET	
1604	SC	Monophasic	CIP, COL, DOX, ENR, FFC, GEN, MAR, NOR, TET	
1607	SC	Monophasic	COL, DOX, ENR, FFC, GEN, MAR, STR, TET	
1593	PR	Monophasic	CEF, CIP, COL, DOX, ENR, FFC, GEN, LSC, MAR, STR, SXT, TET	
1571	MT	Monophasic	CEF, CIP, COL, DOX, ENR, FFC, FOS, GEN, MAR, NOR, STR, SXT, TET	
1573	MG	Monophasic	COL, DOX, ENR, FFC, GEN, LSC, MAR, STR, SXT, TET	
1574	MG	Monophasic	COL, DOX, ENR, FFC, GEN, LSC, MAR, STR, SXT, TET	
1576	MG	Monophasic	CIP, COL, DOX, ENR, FFC, GEN, MAR, NOR, STR, SXT, TET	
1577	MG	Monophasic	CIP, COL, DOX, ENR, FFC, GEN, LSC, MAR, NOR, STR, SXT, TET	
1580	MG	Monophasic	CIP, COL, DOX, ENR, FFC, GEN, MAR, NOR, STR, SXT, TET	
1588	MG	Monophasic	CIP, DOX, ENR, FFC, GEN, MAR, NEO, NOR, STR, SXT, TET	
1594	MG	Monophasic	COL, DOX, ENR, FFC, GEN, MAR, NEO	
1595	MG	Monophasic	CIP, COL, DOX, ENR, FFC, GEN, MAR, NOR, STR, SXT, TET	
1597	SC	Monophasic	CIP, COL, DOX, ENR, FFC, GEN, LSC, MAR, NEO, NOR, STR, SXT, TET	
1598	MT	Monophasic	CIP, COL, DOX, ENR, FFC, GEN, LSC, MAR, NOR, STR, SXT, TET	
1599	MG	Group E4 (O:19:-)	CIP, COL, DOX, ENR, FFC, GEN, LSC, MAR, NEO, STR, SXT, TET	
1572	MG	Monophasic	CEF, CIP, DOX, ENR, FFC, GEN, MAR, NOR, STR, SXT, TET	
1610	SC	Monophasic	COL, DOX, ENR, FFC, GEN, MAR, TET	
1611	SC	Monophasic	CIP, COL, ENR, FFC, GEN, MAR, STR, TET	
1620	RS	Monophasic	ENR, FFC, GEN, MAR, STR, SXT	
1636	SP	Monophasic	CIP, COL, DOX, ENR, FFC, GEN, MAR, NOR, STR, SXT, TET	
1651	PR	Monophasic	CIP, DOX, ENR, FFC, GEN, MAR, NOR, STR, SXT, TET	
1653	MG	Monophasic	CIP, DOX, ENR, FFC, GEN, MAR, NEO, NOR, STR, SXT, TET	
1656	MG	Monophasic	CIP, DOX, ENR, GEN, MAR, NOR, STR, SXT, TET	
1659	MG	Monophasic	CIP, COL, DOX, ENR, FFC, GEN, LSC, MAR, STR, SXT, TET	
1660	MG	Monophasic	CIP, COL, DOX, ENR, FFC, GEN, MAR, NOR, STR, SXT, TET	
1661	MG	Monophasic	CIP, DOX, ENR, FFC, GEN, LSC, MAR, STR, SXT, TET	
1665	MG	Monophasic	CIP, COL, DOX, ENR, FFC, GEN, MAR, NOR, STR, SXT, TET	
1603	SC	Monophasic	CEF, CIP, COL, DOX, ENR, FFC, GEN, MAR, TET	
1677	SC	Monophasic	CIP, COL, ENR, FFC, GEN, MAR, NOR, TET	
1578	MG	Rissen	FFC, NEO	
1591	MG	Rissen	COL, DOX, ENR, FFC, TET	
1600	MG	Rissen	DOX, FFC, NEO, STR, SXT, TET	
1563	MT	Monophasic	COL, DOX, FFC, GEN, STR, TET	
1579	MG	Monophasic	COL, DOX, FFC, GEN, NEO, STR, TET	
1587	SP	Monophasic	COL, DOX, ENR, TET	
1619	SP	Monophasic	COL, DOX, FFC, GEN, STR, TET	
1624	SC	Monophasic	COL, DOX, FFC, GEN, STR, TET	
1630	SC	Monophasic	COL, FFC, GEN, NEO, STR, TET	
1635	SC	Monophasic	COL, FFC, GEN, NEO, STR, TET	
1638	SC	Monophasic	COL, DOX, FFC, GEN, NEO, STR, TET	
1639	SC	Monophasic	COL, DOX, GEN, TET	
1646	SC	Monophasic	COL, DOX, FFC, GEN, NEO, STR, TET	
1667	MG	Monophasic	DOX, ENR, FFC, GEN, MAR, SXT, TET	
1590	MG	Typhimurium	COL, ENR, FFC, GEN, MAR, NEO, SXT, TET	
1584	MG	Monophasic	COL, DOX, TET	
1596	DF	Monophasic	COL, DOX, ENR, FFC, GEN, NEO, STR, TET	
1606	SC	Monophasic	COL, DOX, GEN, TET	
1615	RS	Monophasic	COL, DOX, GEN, TET	
1618	RS	Monophasic	COL, DOX, GEN, NEO, TET	
1621	SC	Monophasic	CEF, COL, DOX, FFC, GEN, NEO, STR, TET	
1622	SC	Monophasic	COL, DOX, FFC, GEN, NEO, TET	
1623	SC	Monophasic	DOX, GEN, TET	
1641	RS	Monophasic	DOX, ENR, GEN, LSC, TET	
1644	SC	Monophasic	COL, FFC, GEN, NEO, TET	
1617	SC	Monophasic	COL, DOX, FFC, GEN, LSC, SXT, TET	
1654	BA	Typhimurium	COL, DOX, FFC, GEN, LSC, SXT, TET	
1655	PR	Typhimurium	DOX, FFC, STR, SXT	
1605	SC	Monophasic	CIP, COL, ENR, FFC, GEN, MAR, SXT, TET	
1616	RS	Monophasic	CEF, COL, SXT, TET	
1642	SC	Monophasic	CIP, COL, DOX, ENR, FFC, GEN, MAR, TET	
1613	SC	Typhimurium	FFC, STR	
1614	RS	Typhimurium	COL, DOX, FFC, STR, TET	
1625	SC	Typhimurium	CEF, COL, FFC, STR	
1626	SC	Typhimurium	COL, FFC, STR	
1643	RS	Typhimurium	COL, FFC, GEN, STR, TET	
1602	SC	Typhimurium	COL, TET	
1632	PR	Typhimurium	COL, SXT, TET	
1637	SP	Monophasic	CIP, COL, DOX, ENR, FFC, GEN, MAR, SXT, TET	
1581	MG	Typhimurium	COL, DOX, TET	
1586	MG	Typhimurium	COL, ENR	
1669	MG	Typhimurium	COL, DOX, FFC, SXT, TET	

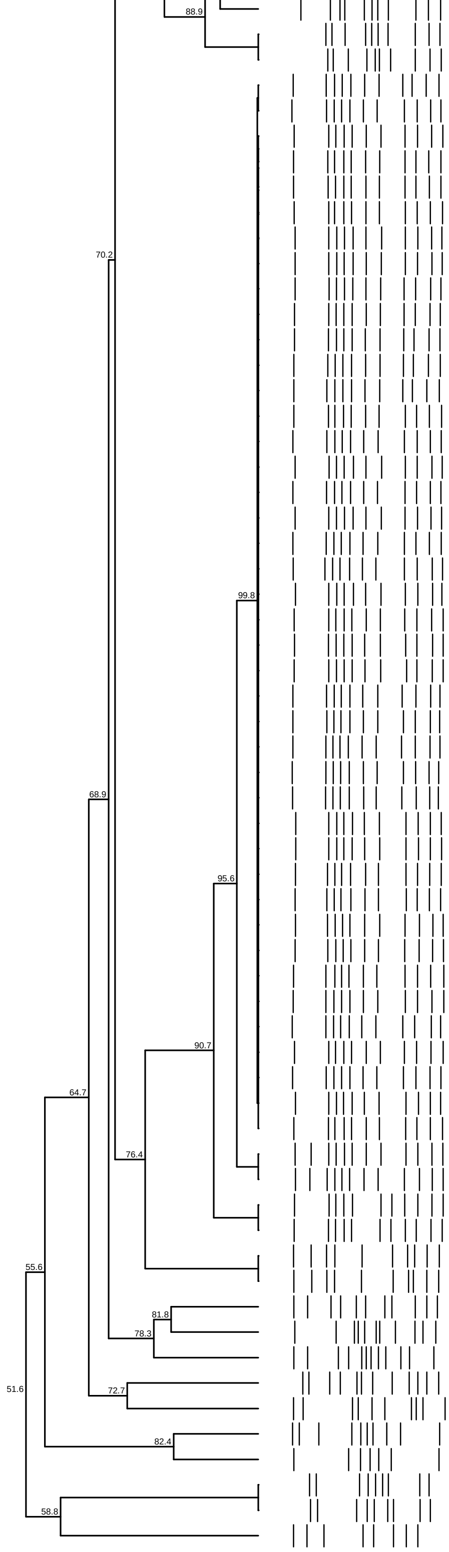

1581	MG	Typhimurium	COL, DOX, TET	
1586	MG	Typhimurium	COL, ENR	
1669	MG	Typhimurium	COL, DOX, FFC, SXT, TET	
1561	MG	Choleraesuis	COL, DOX, FFC, GEN, LSC, STR, TET	X
1566	MG	Choleraesuis	DOX, FFX, GEN, MAR, NEO, STR, TET	
1544	MG	Choleraesuis	DOX, FFC, GEN, STR, TET	
1548	PR	Choleraesuis	DOX, FFC, GEN, STR, TET	
1549	PR	Choleraesuis	DOX, FFC, GEN, STR, TET	
1551	PR	Choleraesuis	DOX, FFC, GEN, TET	
1554	MG	Choleraesuis	DOX, FFC, GEN, STR, TET	
1547	SP	Choleraesuis	DOX, ENR, FFC, GEN, STR, TET	
1556	SP	Choleraesuis	DOX, FFC, GEN, STR, TET	X
1557	SP	Choleraesuis	COL, DOX, FFC, GEN, STR, TET	
1558	MG	Choleraesuis	DOX, FFC, GEN, STR, TET	
1559	MG	Choleraesuis	FFC, GEN, STR, TET	
1560	MG	Choleraesuis	DOX, FFC, GEN, STR, TET	
1550	PR	Choleraesuis	DOX, FFC, GEN, STR, TET	X
1567	MG	Choleraesuis	DOX, FFC, GEN, STR, TET	
1553	SP	Choleraesuis	DOX, FFC, GEN, STR, TET	
1570	MG	Choleraesuis	DOX, ENR, FFC, GEN, STR, TET	
1555	SP	Choleraesuis	DOX, ENR, FFC, GEN, STR, TET	
1575	MG	Choleraesuis	DOX, ENR, FFC, GEN, STR, TET	
1583	GO	Choleraesuis	COL, DOX, ENR, FOS, GEN, MAR, STR, TET	X
1552	SP	Choleraesuis	COL, DOX, FFC, GEN, STR, TET	
1543	MG	Choleraesuis	DOX, FFC, GEN, STR, TET	
1601	RS	Choleraesuis	DOX, FFC, GEN, STR, TET	
1608	SC	Choleraesuis	COL, DOX, TET	
1612	PR	Choleraesuis	COL, FFC, STR, TET	
1627	SC	Choleraesuis	COL, DOX, FFC, GEN, TET	
1628	PR	Choleraesuis	TET	
1631	RS	Choleraesuis	ENR, FFC, GEN, STR	X
1633	SC	Choleraesuis	COL, DOX, GEN, FFC, STR, TET	X
1649	MG	Choleraesuis	COL, DOX, FFC, TET	
1650	PR	Choleraesuis	DOX, FFC, GEN, TET	
1657	MG	Choleraesuis	DOX, FFC, GEN, TET	
1658	MG	Choleraesuis	DOX, FFC, TET	
1673	SC	Choleraesuis	DOX, STR, TET	
1674	SC	Choleraesuis	DOX, GEN, STR, TET	
1675	RS	Choleraesuis	DOX, FFC, GEN, STR, TET	
1676	RS	Choleraesuis	DOX, FFC, GEN, STR, TET	
1565	SC	Choleraesuis	COL, DOX, ENR, FOS, GEN, MAR, STR, TET	
1542	MG	Choleraesuis	DOX, ENR, FFC, GEN, MAR, STR, TET	
1568	MG	Choleraesuis	DOX, ENR, FFC, GEN, NEO, STR, TET	
1647	MG	Choleraesuis	COL, DOX, ENR, FFC, STR, TET	
1640	SC	Choleraesuis	COL, DOX, FFX, GEN, STR, TET	
1546	NI	Choleraesuis	DOX, ENR, FFC, GEN, STR, TET	
1672	SC	Choleraesuis	DOX, NEO	
1545	NI	Choleraesuis	COL, DOX, ENR, FFC, GEN, STR, TET	
1541	NI	Choleraesuis	ENR, NEO	
1652	SP	London	DOX, FFC, GEN, SXT, TET	
1662	MG	London	COL, ENR, FFC, GEN, LSC, STR, SXT, TET	
1634	SC	Anatum	COL, GEN, STR, TET	
1666	MS	Infantis	COL, FFC, TET	
1582	MG	Derby	COL, NEO, TET	
1589	MG	Group D	COL, DOX, TET	
1668	PR	Newport	NONE	
1569	MG	Monophasic	COL, DOX, FFC, SXT, TET	
1592	NI	Bovinsmorbificans	COL	
1564	MG	Panama	COL, DOX, FFC, GEN, LSC, STR, TET	
1664	MG	Panama	COL, DOX, FFC, TET	
1648	MG	Oslo	CEF, COL, ENR, FFC, NEO	
